# Dietary Selenium Nanoparticles Improved Growth and Health Indices in Asian Seabass (*Lates calcarifer*) Juveniles Reared in High Saline Water

**DOI:** 10.1155/2024/7480824

**Published:** 2024-01-10

**Authors:** Hamzeh Mohtashemipour, Takavar Mohammadian, Mansour Torfi Mozanzadeh, Mehrzad Mesbah, Abdolhossein Jangaran Nejad

**Affiliations:** ^1^Department of Livestock, Poultry and Aquatic Animal Health, Faculty of Veterinary Medicine, Shahid Chamran University of Ahvaz, Ahvaz, Iran; ^2^Member of Excellence Center of Warm Water Fish Health, Shahid Chamran University of Ahvaz, Ahvaz, Iran; ^3^South Iran Aquaculture Research Centre, Iranian Fisheries Science Research Institute (IFSRI), Agricultural Research Education and Extension Organization (AREEO), Ahwaz, Iran; ^4^Department of Basic Sciences, Faculty of Veterinary Medicine, Lorestan University, Khorramabad, Iran

## Abstract

A 60-day study was carried out to determine the effect of dietary selenium nanoparticles (SeNP) on growth, digestive enzymes, and health status of Asian seabass (*Lates calcarifer*, 46.5 ± 0.2 g) juveniles reared in high saline water (48 ppt). Five levels of SeNP were added to a basal diet (45% protein, 15% lipid), including control (0), 0.5 (SeNP0.5), 1.0 (SeNP1), 2 (SeNP2), and 4 (SeNP4) mg SeNP kg^−1^ diet. Fish were stocked into fifteen 2,000 L tanks (50 fish tank^−1^) filled with 1,800 L sand-filtered seawater (26.5 ± 1.5°C, 48.0 ± 0.2 ppt) in a flow-through system. Each dietary treatment was performed in three replicates. The growth rate positively increased in both linear and quadratic trends with increasing dietary SeNP level (*P* < 0.05). The liver Se concentration increased with increasing SeNP in diet (*P* < 0.05). Gut total protease, trypsin, chymotrypsin, alkaline phosphatase (ALP), lipase, and *α*-amylase activities were significantly enhanced in the SeNP4 group compared to the other treatments (*P* < 0.05). Antioxidant capacity improved in fish-fed SeNP2 and SeNP4 diets regarding catalase and superoxide dismutase activities and the liver glutathione content. Serum lysozyme and hemolytic activities and white blood cells' respiratory burst activity in the control were lower than in fish-fed SeNP-supplemented diets. Serum total protein, globulin, and globulin/albumin ratio in fish-fed SeNP1, SeNP2, and SeNP4 diets were higher than the other groups (*P* < 0.05). The *interleukin-10* and *granulocyte-macrophage colony-stimulating factor* genes' relative transcription levels in the gut of fish-fed SeNP4 were higher than the other groups. Serum cholesterol, triglycerides, ALP, aspartate aminotransferase, alanine aminotransferase, and lactate dehydrogenase significantly decreased in fish with increasing SeNP content in the diet. In conclusion, supplementing the diet with 4 mg kg^−1^, SeNP was recommended to improve growth and health indices in *L. calcarifer* juveniles reared in high saline water (48 ppt).

## 1. Introduction

Selenium has an integral role in various physiological processes, such as optimal growth, development, and antioxidant defense in fish. It exerts its effects through the synthesis of selenoproteins [1]. Selenoproteins actively contribute to different physiological functions such as antioxidant defense, thyroid hormone synthesis, immune system, and reproduction [2–4]. Selenium-dependent antioxidant enzymes, such as glutathione peroxidase (GPx) and thioredoxin reductase, actively quench the production of free radicals and alleviate oxidative stress [5]. By lowering the levels of hydrogen and lipid peroxides in various cells, these enzymes improve resistance to oxidative damage. The dietary selenium is determined by the activity of these enzymes in the liver or plasma [2, 6]. However, there is a minimum gap between dietary Se requirement and toxicity [7].

The Se form, diet formulation, culture condition, fish species, life stage, health status, and body size can affect the efficacy of dietary Se [8, 9]. Typically, Se can be supplemented in aquafeeds in inorganic (e.g., selenate sodium) or organic (e.g., hydroxy selenomethionine) forms. Due to higher bioavailability, an interest in applying nano trace elements in aquafeeds has increased [8, 9]. Selenium nanoparticles (SeNP) have higher chemical stability, lower toxicity, and high capacity to slowly release Se after ingestion, which enhances the efficiency of selenoprotein synthesis in the body [8–10]. Several studies demonstrated the positive effects of supplementing aquafeeds with SeNP on growth, feed efficiency, and antioxidant capacity during the juvenile stage in various fish species [8, 9]. The deposition effectiveness of SeNP in the medaka (*Oryzias latipes*) diet was shown to be higher than that of sodium selenite [11]. It is also anticipated that the usage of SeNP will involve a considerably smaller quantity as SeNP allows a better control of toxicity and has a higher digestibility and easier assimilation when compared to conventional Se sources. In this context, it has been reported that at the same dietary Se level (0.7 mg kg^−1^), SeNP generates more growth performance, antioxidant defense, and innate immune responses than other Se forms in common carp (*Cyprinus carpio*) [12] and Nile tilapia (*Oreochromis niloticus*) [13]. In addition, SeNP can effectively modulate the immunocompetence of farmed aquatic fish through humoral (e.g., lysozyme (LYZ) activity) and cellular (e.g., respiratory burst activity (RBA)) immune responses as well as immune-related genes (e.g., interleukin 8 and IL-1*β*) [12–20]. Furthermore, the review by Khalil et al. [9] indicated that SeNP could improve hematological parameters (e.g., elimination of anemia, the increment of blood hemoglobin (HB), oxygen-carrying capacity, and the reduction of red blood cell (RBC) hemolysis) and liver health (e.g., modulation of the liver enzymes) in various fish species. Uncertain molecular mechanisms underlie the action of SeNP and its conversion to active selenoproteins. It appears likely that the gut microbiota will transform nanoselenium into selenite, H_2_Se, or Se-phosphate, which will then trigger the production of selenoproteins [21, 22].

Asian seabass (*Lates calcarifer*) has promising characteristics for aquaculture, such as high fecundity, high growth rate, optimal feed conversion ratio, and high tolerance to various culture conditions [23]. Its global production reached 108,000 tons in 2019 [24]. Previous studies in Asian seabass proved that supplementing dietary SeNP not only promoted growth rate but also enhanced its resistance against *Vibrio harvei* [25–27]. Previous research by Mozanzadeh et al. [23] showed that rearing *L. calcarifer* in high saline water (48 ppt) markedly reduced its growth performance, feed conversion ratio, liver GPx activity, gut lipase activity, plasma LYZ, and hemolytic activities (HAs) and plasma total protein level in this species. These adverse effects were associated with the increment of stress indices such as plasma lactate, cortisol, and glucose and an increase in the liver enzymes such as alanine aminotransferase (ALT), aspartate aminotransferase (AST), lactate dehydrogenase (LDH) and alkaline phosphatase (ALP) that was in concomitant with the elevation of malondialdehyde level in the liver [23]. Thus, the current research aimed to evaluate the effects of graded levels of SeNP on growth and health indices of *L. calcarifer* juveniles reared in high saline water (48 ppt).

## 2. Materials and Methods

### 2.1. Experimental Feeds

A basal diet was supplemented with five levels of SeNP, including 0 (Control), 0.5 mg kg^−1^ (SeNP0.5), 1 mg kg^−1^ (SeNP1), 2 mg kg^−1^ (SeNP2), and 4 mg kg^−1^ (SeNP4) (Table 1) according to Khademzadeh et al. [2]. The selected dosages of the SeNP were based on previous studies in other fish species and Asian sea bass [8, 9, 25, 26]. Bovine serum albumin (ALB)-loaded-SeNP (particle size: 30–45 nm; shape: spherical; purity: 99.95%; actual density: 3.89 g cm^3^; Iranian Nanomaterials—Pioneers, Iran, CAS registry number: 7782-49-2) was synthesized by the inclusion of bovine serum ALB to the redox system of selenite and glutathione (GSH) [28]. Dry ingredients were mixed (20 min), and oils were added and blended for 10 min. Finally, the prescribed SeNP dosages were dissolved in distilled water, added to the mixture, and mixed for 10 min to form a soft dough. The dough was subsequently cold pelleted (4 mm) with a meat grinder and dried at room temperature with a fan, and the dried pellets were kept in a freezer (−20°C).

### 2.2. Husbandry

In total, 750 *L. calcarifer* juveniles (46.5 ± 0.2, initial body weight ± standard error) were purchased from a nursery center (Delvar, Iran) and transferred to a commercial marine fish hatchery (Mahshahr, Iran). Fish were stocked into 15 2,000 L rectangular concrete tanks (50 fish/tank) filled with 1,800 L sand-filtered seawater and divided into five treatment groups, with three replicates. Tanks were supplied with seawater in a flow-through system (1 L min^−1^), and based on the water flow rate, about 80% of water was exchanged daily. Fish were fed with the control diet for 14 days to adapt to the husbandry system; then, for 60 days, the experimental feeds were offered to fish thrice daily (08:00, 12:00, and 16:00) up to satiation, ensuring no pellet was left uneaten. Temperature = 26.5 ± 1.5°C, salinity = 48.0 ± 0.2 ppt, pH = 7.9 ± 0.3, and dissolved oxygen = 6.2 ± 0.5 ppm were evaluated once a week. The photoperiod was 12 hr light and 12 hr darkness. The water was supplied from hypersaline bays (45–50 ppt), which exist in the local water bodies of Khuzestan province (northwest of the Persian Gulf), which have high potential for rural aquaculture activities. The choice of water salinity level in the present study was based on the findings of Mozanzadeh et al. [23], who reported the negative effects of high saline water (48 ppt) on the growth, digestive enzymes activity, and health status of *L. calcarifer* juveniles.

### 2.3. Sampling and Selenium Content Analysis

On Day 30 (six fish per tank) and Day 60 (six fish per tank), sampling of fish was done. Fish were fasted a day before each sampling. The biometry of each fish was done individually. The growth and feed efficiency parameters were determined based on the following equations:

Weight gain (WG, %) = ((final weight (g) − initial weight (g))/initial weight (g)) × 100.

Specific growth rate (SGR, %) = ((ln final weight (g) − ln initial weight (g))/*t*) × 100, where *t* is experimental period = 60 days.

Feed conversion ratio (FCR) = feed intake (g)/weight gain (g).

Fulton's condition factor (*K*, %) = (final weight (g)/total length^3^ (cm)) × 100.

Survival = (number of fish in each group remaining on Day 60/initial number of fish) × 100.

For bleeding, six fish from each tank were anesthetized (2-phenoxyethanol, 300 ppm) and bled from the caudal vein. For assessing complete blood count, an aliquot of the blood was transferred into a 500 *µ*L microtube containing anticoagulant (10 *µ*L heparin sodium, 5,000 U) and kept close to a piece of ice (4°C) up to analyses. The rest of the blood was allowed to clot, centrifuged (5,000 *g*, 4°C, 10 min), and then sera were maintained in a −80°C freezer. In addition, the liver of the same fish was dissected on ice (0–4°C), and kept in a −80°C freezer for further evaluation of antioxidant status. The whole gut of the same fish (0–4°C) was dissected on ice, transferred to a cryotube, and kept in a −80°C freezer to assess digestive enzyme activity. In addition, the livers of the three fish per replicate (nine fish per treatment) were dissected and kept in a freezer at −80°C for further evaluation of selenium concentration. The gut of the same fish (three fish per replicate, nine fish per treatment) was snap-frozen in liquid nitrogen, then transferred into a cryotube and kept in a −80°C freezer to evaluate the expression of immune-related genes. The analysis of Se in the experimental diets and liver was performed by ICP-MS (Inductively Coupled Plasma Mass Spectrometry, Agilent 7500, Yokogawa Analytical Systems, Japan) after digesting the samples in acidic condition (HNO_3_ : H_2_O_2_ at 5 : 1) according to standard methods [29, 30].

### 2.4. Digestive Enzymes and Antioxidant Status

The gut samples were homogenized in ice-cold (0−4°C) mannitol buffer (50 mM mannitol + 2 mM tris-HCl, pH 7) at a ratio of 30 : 1 (v/w) for 60 s [31]. The homogenate was centrifuged at 9,000x *g* (10 min at 4°C), then the supernatant was extracted and centrifuged again at 34,000x *g* (30 min at 4°C). The remaining precipitate was suspended in 1 mL of buffer (0.1 M KCl, 5 mM Tris-Hepes, 1 mM DTT; pH 7.5) to evaluate ALP in the gut's brush border [32, 33]. Soluble protein [34] trypsin (E.C. 3.4.21.4) [35], chymotrypsin (EC. 3.4.21.1) [36], protease (EC.3.4.21–24) [37], ALP (E.C. 3.1.3.1) [38], *α*-amylase (E.C. 3.2.1.1) [39], and lipase (E.C. 3.1.1) [40] were determined using standard protocols.

The liver samples were defrosted, weighed, and then homogenized in ratio of 1–9 (w/v) of cold potassium phosphate buffer (0.1 M, pH = 7.4, 4°C) at 10,000x *g* for 60 s. The homogenate was centrifuged (9,000x *g*, 30 min, 4°C); the supernatant was removed and aliquoted, then kept at −80°C. Catalase (CAT) (E.C. 1.11.1.6) [41], superoxide dismutase (SOD) (E.C. 1.15.1.1) [42], and GSH level [43] were determined according to standard methods.

### 2.5. Hematology

RBC, white blood cell (WBC), HB, hematocrit (Hct), mean cell hemoglobin (MCH), the mean cell volume (MCV), and the mean cell hemoglobin concentration (MCHC) were examined by standard methods [44, 45]. RBA [46], LYZ [47], and HAs [48] of serum were determined according to standard methods. Serum total protein (Cat No: 18907), ALB (Cat No: 18901), glucose (Cat No: 18900), triglycerides (Cat No: 18907), calcium (Cat No: 18950), ALT (Cat No: 18872), AST (Cat No: 18878), ALP (Cat No: 18874), and LDH (Cat No: 18888) were determined using commercial diagnostic kits (Pars Azmoon Co., Tehran, Iran) with the following equation: (absorption of sample/absorption of standard) × concentration of standard [49]. The amount of globulin (GLOB) was calculated after subtracting ALB from total protein content. The expected ranges from the kit for the biochemical parameters were as follows: total protein (0.5–15 g dL^−1^), ALB (0.2–6 g dL^−1^), glucose (5–400 mg dL^−1^), triglycerides (5–700 mg dL^−1^), calcium (0.4–25 mg dL^−1^), ALP (3–858 U L^−1^), ALT (0.5–300 U L^−1^), AST (0.5–300 U L^−1^), and LDH (5–3,000 U L^−1^).

### 2.6. Gene Transcription

Total RNA was extracted by TRIzol (Invitrogen, Carlsbad, CA, USA) based on the manufacturer's instruction and kept at −80°C. The level and purity of extracted RNA were determined by a spectrophotometer (NanoDrop Technologies, Wilmington, USA) and agarose gel (1%). cDNA synthesis was carried out by employing an iScript cDNA Synthesis Kit (Bio-Rad CA, USA), and the polymerase chain reaction was performed in duplicate for each sample with the SYBRgreen method in an iQ5 iCycler thermal cycler (Bio-Rad). The sequences of specific primers used for determining the transcription levels of *interleukin-10 (IL-10*) and *granulocyte-macrophage colony-forming cells (GMCFC*) in the gut of fish are presented in Table 2. The reactions were conducted according to Abbaszadeh et al. [50]. *β*-Actin was used as a reference gene in each sample. The iQ5 optical system software (ver. 2.0) was used to analyze the obtained data.

### 2.7. Statistics

All data were presented as means ± standard error of the mean of three replicates. Statistical analysis was carried out using one-way ANOVA (SPSS 23.0, Chicago, IL, USA) followed by Tukey's comparison of means. All data were also subjected to polynomial orthogonal regression analyses to detect the potential linear or quadratic influence of dietary SeNP on the physiological responses. In all cases, *P* < 0.05 was considered as significant.

## 3. Results

### 3.1. Growth Performance and SeNP Concentration

**Figure 1:**
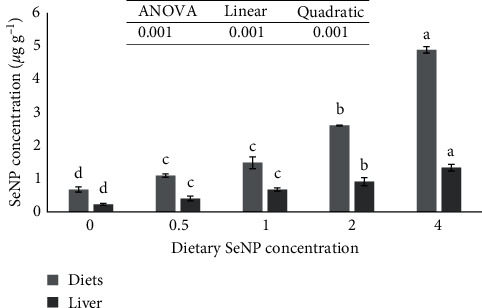
Actual selenium (Se) concentrations in the diets and liver of Asian seabass (*Lates calcarifer*) juveniles fed basal diet supplemented by various concentrations of selenium nanoparticle (SeNP). Data are mean ± SE (*n* = 3). For each bar, values with different letters show significant differences (*P* < 0.05).

Survival rate was 100% in all groups (Table 3). Fish fed with SeNP4 had higher final weight, weight gain, and specific growth rate than the other groups (*P* < 0.05). The growth performance positively increased in both linear and quadratic trends with increasing the SeNP level in the basal diet (*P* < 0.001). In the final length, Fulton's condition factor and FCR were not affected by the inclusion of SeNP in the diet. The amount of Se increased in the liver with increasing SeNP concentration (*P* < 0.001, Figure 1).

### 3.2. Digestive Enzymes

In the middle of the feeding trial (Day 30), total protease and ALP activities in the SeNP4 group were higher than those of other treatments (Table 4). In addition, trypsin, chymotrypsin, and lipase activities in SeNP2 and SeNP4 were higher than the other groups. *α*-Amylase activity in SeNP4 was higher than in the other groups. The control group had the lowest values of digestive enzyme activities compared to fish-fed SeNP-supplemented diets.

At the end of the feeding trial (Day 60), fish-fed SeNP4 had the highest total protease, trypsin, ALP, lipase, and *α*-amylase activities, but the lowest values were in the control group (*P* < 0.05). Chymotrypsin activity in fish-fed SeNP-supplemented diets was higher than in the control. Digestive enzyme activities positively increased in both linear and quadratic trends with increasing SeNP levels (*P* < 0.001).

### 3.3. Antioxidant Capacity

After 30 days, the highest GSH level in the liver was in fish-fed SeNP2 and SeNP4 diets, and the control group had the lowest value (Table 5). In the serum, the GSH level in the SeNP4 group was higher than the others. CAT activity in the liver of fish-fed SeNP2 and SeNP4 diets was higher than others, but in the serum, the lowest CAT activity was in the control group (*P* < 0.05). SOD activity in the liver of fish-fed SeNP4 was more than other treatments, but this group showed lower serum SOD activity than other groups after 30 days.

At the end of the feeding trial (Day 60), the GSH levels in the liver of fish-fed SeNP2 and SeNP4 diets were higher than in other treatments, and the control group had the lowest GSH level. The control group had higher serum GSH levels than the fish-fed SeNP2 diet. CAT activity in the liver of fish-fed SeNP2 was higher than other treatments. CAT activity in the serum of the control group was lower than in fish-fed SeNP-supplemented diets. SOD activity in the serum and liver of fish-fed SeNP2 and SeNP4 diets was higher than in the other groups. Antioxidant factors in the liver of fish positively increased in both linear and quadratic trends with increasing SeNP levels at both sampling times (*P* < 0.001).

### 3.4. Immune Responses

In the middle of the feeding trial (Day 30), the serum LYZ and HAs and total protein content in fish-fed-SeNP-supplemented diets were higher than in the control (Table 6). The serum ALB level in the SeNP4 was lower than in the other treatments (*P* < 0.05). The serum globulin in SeNP2 and SeNP4 was higher than the others. The serum GLOB/ALB ratio in the SeNP4 diet was higher than in the other groups. Serum RBA was not affected by dietary Se level (*P* > 0.05).

At the end of the experiment (Day 60), SeNP4 had the highest serum LYZ activity compared to the other treatments. Serum HA, total protein, GLOB, and GLOB/ALB ratio in fish-fed SeNP1, SeNP2, and SeNP4 diets were higher than the others (*P* < 0.05). Serum RBA activity in the SeNP2 group was higher than in other groups.

### 3.5. Hematology

In the middle of the feeding trial (Day 30), blood HB content in fish-fed SeNP-supplemented diets was higher than in the control group (Table 7). Blood Hct and MCV levels in fish-fed SeNP0.5 were higher than the other treatments (*P* < 0.05). The MCHC level in fish-fed SeNP1 and SeNP2 diets was higher than the control and SeNP0.5 groups. The values of the RBC, WBC, and MCH were not affected by dietary Se level (*P* > 0.05).

At the end of the experiment (Day 60), the RBC count in the control group was higher than in fish-fed SeNP-supplemented diets. Fish-fed SeNP4 had the highest Hct level. The MCV values in fish fed the control and SeNP4 diets were higher than the SeNP2 group (*P* < 0.05). WBC, HB, and MCH values were not affected by the experimental diets.

### 3.6. Biochemical Indices

Serum glucose and calcium levels were not changed among the treatments on both Day 30 and Day 60 (Table 8). In the middle of the feeding trial (Day 30), serum cholesterol, triglycerides, AST, and LDH gradually decreased with increasing SeNP levels in the diet and showed both linear and quadratic trends (*P* < 0.05). Serum ALP level in fish-fed SeNP4 was lower than in fish-fed SeNP1 and SeNP2 diets, but serum ALT level did not change after 30 days among various treatments.

At the end of the feeding trial (Day 60), serum cholesterol, triglycerides, AST, ALP, and LDH gradually decreased with increasing SeNP levels in the diet. Serum ALT level gradually decreased with increasing SeNP concentration in the diet, and it showed both linear and quadratic trends on Day 60.

### 3.7. Immune-Related Genes

**Figure 2:**
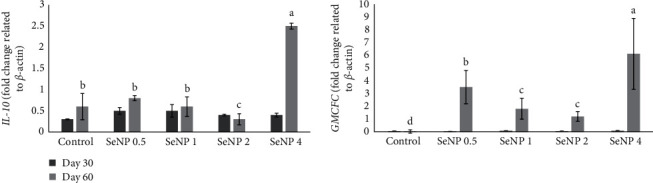
Immune-related genes, including *interleukin-10 (IL-10*) and *granulocyte-macrophage colony-forming cells (GMCFC*), the gut of *L. calcarifer* juveniles fed the experimental diets at Days 30 and 60. Bars with a common superscript letter are not significantly different from the other dietary groups (*P*  > 0.05). Data are presented as the mean ± SE of three replicates.

The gut immune-related genes, including *IL-10* and *GMCFC*, were not affected by the experimental diets at Day 30 (*P* > 0.05, Figure 2). However, at Day 60, the *IL-10* and *GMCFC* genes' relative transcription levels in the gut of fish-fed SeNP4 were higher than the other groups (*P* < 0.05).

## 4. Discussion

### 4.1. Growth

The suitable amount of dietary Se is species-specific and varies with the source/form ingested, its bioavailability, the levels of polyunsaturated fatty acids, vitamin E, Se, and the other antioxidants in the aquafeed [9]. SeNP has a large surface but nanosize that enhances its permeability and bioavailability in the fish body compared to other Se forms [51]. It was proved that there is a strong relationship between growth rate and the selenoproteins gene transcription level in fish [52]. Selenomethionine, as the central part of organic Se, is retained as selenoprotein and plays a role in protein synthesis and cellular growth [6, 53]. In addition, Se is an integral part of the deiodinase enzyme that bioconverts thyroxin to triiodothyronine and profoundly affects the synthesis and release of growth hormones [2]. Moreover, Se, as the central part of GPx, provides high antioxidant capacity and protects cell membranes against reactive oxygen species and consequently can improve growth rate in farmed aquatic species [9]. Finally, the positive effects of SeNP on fish growth are possibly related to the vital role of Se in selenoproteins synthesis in the gut epithelial cells that cause a positive impact on the intestinal histomorphological features such as increasing the integrity, length, and width of the gut villi that consequently enhance nutrient absorption [54]. In the present study, supplementation of diet with 4 mg kg^−1^ SeNP significantly enhanced the growth rate in *L. calcarifer* juveniles and was associated with the increment of the liver Se concentration, digestive enzyme activity, antioxidant capacity and immune responses. In this context, it has been proved that supplementing a soybean protein-rich diet with 0.2% organic Se enhanced growth in *L. calcarifer*, which was attributed to better FCR and increased muscle Se in this species [26]. Furthermore, supplementing the diet with 4 mg kg^−1^ SeNP enhanced growth in *L. calcarifer* reared in freshwater and was associated with better feed intake and higher liver Se concentration in this species [26]. Also, a 5%–10% replacement of dietary fish meal with selenium-enriched Spirulina was possible without any adverse effect on the growth rate of *L. calcarifer* correlated with the increment of GPx activity in serum [27]. Supplementing the diet with SeNP also promoted growth rate in other marine fish species such as gilthead seabream (*Sparus aurata*) [55], red bream (*Pagrus major*) [17], European seabass (*Dicentrarchus labrax*) [19, 20], and yellowfin seabream (*Acanthopagrus arabicus*) [56].

The Se level in the liver mirrors a more distinct dose–response to dietary Se because of its role in the detoxification of external agents and the regulation of Se level in the entire body [17]. In the present study, Se concentration in the liver increased with increasing SeNP dosage in the diet, as also reported in other fish species such as common carp [57], red seabream [17], Arabian seabream [4], Striped catfish (*Pangasianodon hypophthalmus*) [58].

### 4.2. Digestive Enzymes

Selenium performs as a pioneer for selenoproteins synthesized in the gut, leading to an enhanced activity of digestive enzymes. Selenium acts as a coenzyme for digestive enzyme synthesis, so the increment of its availability can increase digestive enzyme activity [59]. It should be mentioned that increasing the integrity of gut villi epithelium [54] may enhance the activity of the villous brush border enzymes such as ALP, as also detected in the current research in the SeNP4 group. In the present study, digestive enzyme activities increased with the increment of dietary SeNP, resulting in a higher growth rate in fish-fed SeNP4. The increment of digestive enzyme activities can elevate nutrient digestibility and provide higher essential nutrients for assimilation through the gut epithelial cells. Also, supplementing the diet with SeNP increased protease activity in red seabream (1 mg kg^−1^) [17] and lipase activity in juvenile Nile tilapia (8 mg kg^−1^) [60]. Furthermore, protease, amylase, and lipase activities were significantly enhanced by supplementing diet with 0.5 [61] and 1.0 mg kg^−1^ SeNP in whiteleg shrimp (*Penaeus vannamei*) and Nile tilapia [62]. Moreover, dietary organic Se (0.2 g kg^−1^) supplementation enhanced amylase and protease activity in marron (*Cherax cainii*) [63]. In contrast, supplementing the diet with 4 mg kg^−1^, SeNP did not have any significant effects on the digestive enzymes of *L. calcarifer* reared in freshwater [64]. These differences in findings of our study with previous research may related to the culture condition, water salinity, and biochemical composition of feed, among other factors.

### 4.3. Antioxidant Capacity

Selenium exerts its antioxidative effects through selenoproteins synthesis, which is an integral part of the active center of GPx, which can protect cell membranes and other threatened organelles by neutralizing peroxide and hydroperoxide radicals [65]. It has been confirmed that SeNP has more antioxidative potential than other Se forms [12, 56] because it could upregulate the expression of GPx by forming selenophosphate [66] and activate the cascade of antioxidant enzymes [8]. SOD by dismutation of the O^2−^ into H_2_O_2_ and H_2_O and CAT by decomposing H_2_O_2_ into O_2_ and H_2_O, respectively, are two vital components of the enzymatic antioxidant defense [67]. GSH is a nonenzymatic antioxidant with a high concentration of sulfhydryl (thiol) groups that is essential for maintaining the intracellular oxidoreductive balance by detoxifying organic hydroperoxides and scavenging reactive oxygen species (like hydrogen peroxide in conjunction with GPx) [67]. In the present study, antioxidant parameters almost increased in the serum and liver of fish-fed SeNP-supplemented diets, suggesting antioxidant capacity boosted in these groups. Similarly, a 5%–10% substitution of dietary fish meal with selenium-enriched Spirulina increased serum GPx activity but did not affect CAT activity in this species [27]. In contrast, supplementing the diet with 4 mg kg^−1^, SeNP did not affect antioxidant enzyme activities but significantly reduced lipid peroxidation levels in *L. calcarifer* reared in freshwater [26]. Other studies also confirmed that SeNP increased antioxidant capacity in gilthead seabream [55], meager (*Argyrosomus regius*) [16], common carp [12, 57], red seabream [17], striped catfish [58], Nile tilapia [68], and zebrafish (*Danio reiro*) [69].

### 4.4. Immunocompetence

Selenium has a positive influence on the immunocompetence of fish by boosting-up the antioxidant capacity [14, 70, 71], antistress effects [14], the regulation of cell signaling molecules (i.e., cytokines) [72], and the regulation of thyroid hormones metabolism through type 2 deiodinase activity [73]. In addition, Le and Fotedar [74] suggested that Se increases the immunocompetence of fish by increasing lymphocyte protein synthesis, which in turn increases the activity of immune cells. LYZ activity can hydrolyze the peptidoglycan layer of Gram-positive bacteria. In addition, by complement system-mediated opsonization system, LYZ can exsert its lytic activity against Gram-negative bacteria [75]. Furthermore, LYZ activates phagocytes and the complement system because it is an opsonin [75]. In the present study, immune responses and immune-related genes of fish improved in fish fed the SeNP-supplemented diet, which coincided with increasing antioxidant capacity and growth rate, especially in fish fed the SeNP4 diet. Also, supplementing diet with 4 mg kg^−1^ elevated serum LYZ activity but did not affect serum globulin content and HA in *L. calcarifer* reared in freshwater [64]. Similarly, supplementing the diet with Se-yeast and SeNP significantly increased serum total protein, globulin, and ALB, as well as increased LYZ, HA, and RBA in meager [16] and common carp [12], respectively. Other studies also reported that supplementing the diet with SeNP increased serum LYZ and HAs in various farmed fish species such as mahseer fish (*Tor putitora*) [76], rainbow trout [69], *Piaractus mesopotamicus* [15], Nile tilapia [67], and red seabream [17].

The blood total protein is a reliable index to demonstrate fish immunocompetence condition [77]. In this study, fish-fed SeNP-supplemented diets had higher amounts of serum total protein and GLOB, indicating its positive influences on the immune status of the *L. calcarifer* as reported in other fish species fed SeNP-supplemented diets [17, 19, 20, 57, 67].

It has been suggested that dietary Se can modulate fish immune responses by inhibiting the release of proinflammatory cytokines and stimulating the release of anti-inflammatory cytokines [9]. In the current research, the upregulated *IL-10* and *GMCFC* genes revealed the immunostimulatory role of SeNP on *L. calcarifer*, which has also been reported in other species, such as European seabass [19, 20]. In grass carp (*Ctenopharyngodon idella*), supplementing a high-fat diet with SeNP significantly down-regulated the expression of proinflammatory genes (*IL−6, IL−8, IL-1β, interferon-γ, and tumor necrosis factor-alpha, TNF-α*) in the gut. It alleviated the oxidative damage of ROS on the gut [78]. Also, Al-Deriny et al. [18] reported that dietary SeNP supplementation upregulated *TNF-α* in Nile tilapia. Moreover, Abd El-Kader et al. [19, 20] reported that dietary SeNP significantly enhanced serum total protein and globulin and increased humoral (i.e., *LYZ* activity) and cellular (i.e., phagocytosis activity) immune responses in European sea bass that were associated with upregulation of *IL-8* and *IL-1β* in the liver.

### 4.5. Hematology

Evaluating hematological factors can be used for examining fish health status, and these parameters are susceptible to nutrition, rearing conditions, water quality, stress, or diseases [79]. Se is essential for hematopoiesis by altering the receptors of transferrin on the hematopoietic tissues and increasing the stability and integrity of blood cells in fish due to its potent antioxidant property that protects blood cells from hemolysis and enhances their life span [76, 80, 81]. In this regard, Le et al. [70] reported that dietary Se-yeast increased GPx activity in RBC's of yellowtail kingfish (*Seriola lalandi*). Previous studies demonstrated the potent antioxidant capacity of the SeNP compared to other Se forms to increase the endurability of RBC membranes by protecting them against membrane damage due to ROS and anemia [12, 13]. In the present study, hematological parameters had some fluctuations; however, SeNP positively increased HB content after 30 days and enhanced Hct in fish fed 4 mg Se kg diet^−1^, suggesting its positive effects on the hematological health of *L. calcarifer*. In this regard, supplementing the diet with 3.5–5.5 mg kg^−1^ organic Se enhanced Hct and GPx activity in *L. calcarifer*, but higher levels (6.5–8.5 mg kg^−1^) reduced their values due to toxicity of Se at higher concentrations [25]. Previous studies demonstrated positive effects of SeNP by increasing hematological factors (e.g., HB, Hct, RBC count) that were mainly associated with the increment of GPx activity in various fish species such as mahseer [76], common carp [12], red seabream [17], European seabass [19, 20] and *Labeo rohita* [82]. Furthermore, Neamat-Allah et al. [13] reported that dietary SeNP protected erythrocytes from hemolysis after challenging *Streptococcus iniae* and prohibited anemia due to subsequent hemorrhagic septicemia in Nile tilapia.

### 4.6. Serum Biochemical Factors

In the current research, supplementing the diet with SeNP significantly reduced cholesterol and triglycerides, suggesting the hypolipidemic effects of this additive on *L. calcarifer*. Selenium supplementation can reduce cytosolic malic enzyme activity that produces nicotinamide adenine dinucleotide phosphate, a substance used to metabolize fatty acids and cholesterol [83]. Therefore, it is likely that higher dietary selenium levels can decrease the malic cytosolic enzyme activity that reduces the amount of NADPH needed for fatty acids and cholesterol synthesis. Furthermore, dietary Se can downregulate the *3-hydroxy 3-methylglutaryl coenzyme A (HMG-CoA) reductase* gene expression [84, 85] that results in the reduction of serum CHO in rats [86]. Likewise, supplementing the diet with SeNP reduced cholesterol and triglycerides in common carp [57] and red seabream [17].

In fish, the elevation of liver enzymes in serum could indicate liver damage or malfunction [87]. In our research, liver enzymes decreased with increasing dietary SeNP levels, and the fish-fed SeNP4 diet showed lower values than other groups, indicating the health-promoting effects of SeNP on the liver. In this regard, Hao et al. [88] reported that LDH and AST were decreased in loach (*Paramisgurnus dabryanus*), receiving 0.39–0.50 mg Se kg^−1^. Also, supplementing the diet with 4 mg kg^−1^ significantly reduced serum ALP, LDH, AST, and ALT in *L. calcarifer* reared in freshwater [64]. Dietary fish meal sparing with 5% selenium-enriched Spirulina reduced serum ALT and AST associated with the increment of GPx activity in *L. calcarifer* [27]. Furthermore, Neamat-Allah et al. [13] reported that dietary SeNP significantly reduced serum ALP, ALT, AST, and LDH levels in Nile tilapia after bacterial challenge compared to a group fed inorganic Se-supplemented diet. In contrast, supplementing the diet with SeNP (2 mg kg^−1^) increased serum ALT and AST in common carp due to the toxic influence of this additive at high inclusion level [57]. It should be mentioned that the consequences of increasing the NPs application in aquaculture, such as the delivery of feed supplements and nutraceuticals, microbial disinfectant, or a treatment method for aquaculture effluents, are the risk of creating a new generation of waste known as nanowastes and the ecotoxicity of the NPs will require more studies to reach a sustainable approach, effective policies and guidelines for the safer usage of NPs in the aquaculture industry [89, 90].

## 5. Conclusion

In summary, the findings of this study revealed that supplementing the basal diet with 4 mg kg^−1^ SeNP significantly increased the growth rate in *L. calcarifer* reared in high saline water (48 ppt), mainly associated with the promotion of digestive enzyme activities and health indices in this species. In addition, better immunocompetence in a fish-fed SeNP-supplemented diet was concomitant with the upregulation of the gut immune-related genes. Moreover, SeNP had hypolipidemic effects on *L. calcarifer* that may decrease the incidence of fatty liver and can improve liver health. Further studies by applying cutting-edge molecular approaches are needed to determine the exact mode of action of SeNP on the growth and immune-related genes and translation of the related proteins in *L. calcarifer* by considering stressful and bacterial challenges.

## Figures and Tables

**Table 1:** Formulation (g/kg) and proximate composition (%) of experimental diets.

Ingredients^a^	Basal diet
Fish meal^b^	455
Soybean meal^c^	80
Corn gluten^c^	80
Wheat gluten^c^	80
Poultry meal^d^	145
Wheat middling	52
Beef gelatin	10
DL-methionine	1
L-lysine	2
Fish oil^b^	20
Soybean oil^c^	20
Soy lecithin^e^	20
Vitamin premix^i^	10
Mineral premix ^j^	10
L-ascorbic acid (50%)^k^	5
Dicalcium phosphate	10
*Proximate composition* (%)	
Moisture	9.0
Crude protein	45.7
Crude lipid	15.1
Ash	9.8

^a^Composition of ingredients as % dry-weight basis (fish meal (60.5% crude protein, 18.0% crude lipid); soybean meal (41% crude protein, 4.2% crude lipid); corn gluten (71.4% crude protein, 4.1% crude lipid); wheat gluten (53.3% crude protein, 2.8% crude lipid); poultry meal (51.2% crude protein, 15.5% crude lipid), gelatin (85% crude protein, crude lipid, 4.2); wheat middling (12% crude protein, 3.0% crude lipid)). ^b^Parskilka Mazandaran, Iran (Clupeonella sp.). ^c^Product of Kesht Va Sanat Shomal Vegetable Oil Factories Complex (Neca, Iran). ^d^Nazdaneh Sepahan, Isfahan, Iran. ^e^Behpak indastrial company, Behshahr, Mazandaran, Iran. ^f^Vitamin premix (IU/kg of premix): Ascorbic acid, 350,000; Retinol, 1,000,000,000; Cholecalciferol, 500,000,000; Tocopherols, 500,000; Vitamin K_3_, 960,000; thiamin, 980,000; riboflavin, 800,000; pyridoxine, 990,000; folic acid, 950,000; cobalamin, 10,000; biotin, 20,000; Niacin, 995,000; pantothenic acid, 980,000. ^g^Mineral premix mg/kg of premix: magnesium, 6,400; copper, 2,000; ferrous, 11,000; zinc, 100; iodine, 300; cobalt, 50; natrium, 5,000. ATA Company, Tabriz, Iran. ^h^Rooyan Darou, Semnan, Iran.

**Table 2:** Primer sequences and amplification efficiencies.

Gene name	Sequences of primers	Accession number	Length	Efficiency (%)
*IL-10*	Forward: CCAATGTGCAACAACCAGTG Reverse: TTCGACGGTCTGATCTAGCA	XM_018686737.1	149	97

*GMCFC*	Forward: ACCCTCTGCCCCAGTTCTTC Reverse: TCTGAGCCAGTGTGGTTGC	XM_035655954.1	115	97

*β-actin*	Forward: CACAGCTAACGGATTCACTCTG Reverse: TTCCATGGCTGAACTTTGGG	XM_018667666.1	134	97

Abbreviations: IL-10, interleukin 10; GMCFC, granulocyte-macrophage colony-forming cell.

**Table 3:** Influence of different levels of selenium nanoparticles dietary supplementation on growth performance of Asian seabass (*Lates calcarifer*).

Parameters	Control	SeNP 0.5	SeNP 1	SeNP 2	SeNP 4	Pooled SE	ANOVA	Linear	Quadratic
IBW (g)	46.8	46.5	46.8	47.6	46.8	0.2	0.547	0.695	0.489
IBL (cm)	15.0	14.8	14.9	14.8	14.8	0.1	0.153	0.890	0.374
FBW (g)	172.3^b^	170.7^b^	173.1^b^	179.0^b^	205.6^a^	4.6	0.001	0.001	0.001
FBL (cm)	23.1	23.3	23.2	22.7	23.5	0.3	0.170	0.952	0.823
WG (%)	268.2^b^	267.1^b^	269.9^b^	275.9^b^	339.7^a^	10.9	0.001	0.001	0.001
SGR (% IBW day^−1^)	2.17^b^	2.16^b^	2.18^b^	2.20^b^	2.47^a^	0.1	0.005	0.001	0.001
K (%)	1.4	1.35	1.38	1.50	1.58	0.0	0.070	0.442	0.87
FCR	1.62	1.67	1.63	1.60	1.54	0.1	0.980	0.530	0.817
Survival (%)	100	100	100	100	100	0.0	1.000	1.000	1.000

The values were expressed as mean ± pooled SE (*n* = 3 tanks). For each parameter, superscript letters denote significant (*P* < 0.05) differences between the values in each row. Abbreviations: IBW, initial body weight; IBL, initial body length; FBW, final body weight; FBL, final body length; WG, weight gain; SGR, specific growth rate; K, Fulton's condition factor; FCR, feed conversion ratio.

**Table 4:** Influence of different levels of selenium nanoparticles dietary supplementation on digestive enzymes (U mg protein^−1^) of Asian seabass (*Lates calcarifer*) (*n* = 6).

Parameters	Sampling time (day)	Control	SeNP 0.5	SeNP 1	SeNP 2	SeNP 4	Pooled SE	ANOVA	Linear	Quadratic
Total protease	30	75.5^e^	141.9^d^	265.8^c^	367.2^b^	620.5^a^	35.6	0.001	0.001	0.001
	60	76.1^e^	329.9^d^	463.5^c^	587.7^b^	650.4^a^	38.1	0.001	0.001	0.001

Trypsin	30	5.7^c^	7.9^b^	8.1^b^	9.5^a^	10.3^a^	0.1	0.001	0.001	0.001
	60	5.7^d^	12.5^c^	13.6^bc^	16.8^b^	26.7^a^	0.3	0.001	0.001	0.001

Chymotrypsin	30	0.2^c^	0.4^bc^	0.6^b^	0.8^a^	0.8^a^	0.0	0.001	0.001	0.001
	60	0.2^b^	0.6^a^	0.7^a^	0.8^a^	0.8^a^	0.1	0.001	0.001	0.001

Alkaline phosphatase	30	4.4^d^	6.5^c^	7.6^b^	8.3^b^	9.1^a^	0.3	0.001	0.001	0.001
	60	4.6^d^	9.5^c^	13.3^b^	15.8^b^	22.7^a^	1.1	0.001	0.001	0.001

Lipase	30	86.8^c^	97.3^b^	98.8^b^	105.4^a^	111.6^a^	0.8	0.001	0.001	0.001
	60	88.4^e^	178.2^d^	206.1^c^	236.9^b^	296.4^a^	12.8	0.001	0.001	0.001

*α*-Amylase	30	0.9^b^	1.0^b^	1.0^b^	1.0^b^	1.2^a^	0.0	0.001	0.001	0.001
	60	0.9^b^	2.0^ab^	2.5^ab^	2.3^ab^	3.3^a^	0.2	0.001	0.001	0.001

The values were expressed as mean ± pooled SE (*n* = 6). For each parameter, superscript letters denote significant (*P* < 0.05) differences between the values in each row.

**Table 5:** Influence of different levels of selenium nanoparticles dietary supplementation on the liver and serum glutathione and antioxidant enzymes of Asian seabass (*Lates calcarifer*) (*n* = 6).

Parameters	Sampling time (day)	Control	SeNP 0.5	SeNP 1	SeNP 2	SeNP 4	Pooled SE	ANOVA	Linear	Quadratic
Liver										

GSH (nmol g^−1^)	30	11.6^c^	14.6^b^	15.6^b^	25.5^a^	22.6^a^	1.0	0.001	0.001	0.001
	60	13.2^c^	26.3^b^	27.3^b^	38.7^a^	38.5^a^	2.0	0.001	0.001	0.001
CAT (U mg protein^−1^)	30	118.7^c^	128.5^c^	167.2^b^	228.1^a^	230.9^a^	8.9	0.001	0.001	0.001
	60	120.6^d^	167.3^c^	175.2^c^	348.7^a^	302.6^b^	16.2	0.001	0.001	0.001
SOD (U mg protein^−1^)	30	35.6^c^	43.3^b^	45.6^b^	45.7^b^	50.5^a^	1.0	0.001	0.001	0.001
	60	37.6^c^	52.4^b^	52.8^b^	63.9^a^	60.7^a^	1.9	0.001	0.001	0.001

Serum										

GSH (nmol mL^−1^)	30	9.4^b^	7.5^b^	9.7^b^	9.0^b^	22.9^a^	1.2	0.001	0.001	0.001
	60	25.3^a^	21.0^ab^	19.0^ab^	15.4^b^	20.5^ab^	1.1	0.068	0.241	0.041
CAT (U mg protein^−1^)	30	147.1^c^	184.7^a^	196.2^a^	168.1^b^	187.2^a^	11.6	0.001	0.204	0.027
	60	41.8^b^	78.0^a^	72.4^a^	65.8^ab^	87.6^a^	4.0	0.001	0.001	0.001
SOD (U mg protein^−1^)	30	34.0^a^	33.3^a^	38.9^a^	34.0^a^	20.0^b^	1.4	0.001	0.117	0.017
	60	26.4^b^	25.6^b^	36.1^ab^	38.9^a^	42.4^a^	1.7	0.001	0.001	0.001

The values were expressed as mean ± pooled SE (*n* = 6). For each parameter, superscript letters denote significant (*P* < 0.05) differences between the values in each row. Abbreviations: GSH, glutathione; CAT, catalase; SOD, superoxide dismutase.

**Table 6:** Influence of different levels of selenium nanoparticles dietary supplementation on blood immune parameters and gut immune-related genes of Asian seabass (*Lates calcarifer*) (*n* = 6).

Parameters	Sampling time (day)	Control	SeNP 0.5	SeNP 1	SeNP 2	SeNP 4	Pooled SE	ANOVA	Linear	Quadratic
LYZ (U mL^−1^)	30	268.6^b^	652.4^a^	621.1^a^	692.6^a^	722.9^a^	35.8	0.001	0.001	0.001
	60	278.3^c^	827.2^b^	933.3^b^	744.4^b^	1458.6^a^	52.8	0.001	0.001	0.001

HA (AU)	30	9.3^b^	12.0^a^	13.8^a^	13.3^a^	11.0^a^	0.3	0.001	0.619	0.001
	60	10.2^c^	15.7^b^	17.0^a^	19.0^a^	19.8^a^	0.7	0.001	0.001	0.001

RBA (OD540)	30	0.32	0.34	0.29	0.28	0.26	0.0	0.252	0.158	0.056
	60	0.18^b^	0.26^ab^	0.22^ab^	0.36^a^	0.29^ab^	0.0	0.001	0.041	0.103

TP (g dL^−1^)	30	2.8^b^	3.2^a^	3.4^a^	3.9^a^	3.5^a^	0.1	0.001	0.003	0.001
	60	2.5^c^	3.6^b^	4.8^a^	5.5^a^	5.5^a^	0.2	0.001	0.001	0.001

ALB (g dL^−1^)	30	1.4^a^	1.6^a^	1.5^a^	1.5^a^	1.2^b^	0.1	0.007	0.018	0.007
	60	1.3	1.6	1.5	1.5	1.8	0.1	0.090	0.057	0.089

GLOB (g dL^−1^)	30	1.4^c^	1.6^c^	1.9^b^	2.4^a^	2.3^a^	0.1	0.001	0.001	0.001
	60	1.2^c^	2.0^b^	3.3^a^	4.0^a^	3.7^a^	0.2	0.001	0.001	0.001

GLOB/ALB	30	1.0^d^	1.0^d^	1.3^c^	1.6^b^	1.9^a^	0.1	0.001	0.001	0.001
	60	0.9^b^	1.3^b^	2.2^a^	2.7^a^	2.1^a^	0.2	0.001	0.050	0.001

The values were expressed as mean ± pooled SE (*n* = 6). For each parameter, superscript letters denote significant (*P* < 0.05) differences between the values in each row. Abbreviations: LYZ, lysozyme; HA, hemolytic activity; RBA, respiratory burst activity; TP, total protein; ALB, albumin; GLOB, globulin; IL-10, interleukin-10; GMCFC, granulocyte-macrophage colony-forming cell.

**Table 7:** Influence of different levels of selenium nanoparticles dietary supplementation on hematological parameters of Asian seabass (*Lates calcarifer*) (*n* = 6).

Parameters	Sampling time (day)	Control	SeNP 0.5	SeNP 1	SeNP 2	SeNP 4	Pooled SE	ANOVA	Linear	Quadratic
RBC (×10^6^ *µ*L)	30	1.8	1.7	2.1	2.1	1.8	0.2	0.078	0.994	0.074
	60	2.7^a^	2.5^b^	2.4^b^	2.4^b^	2.4^b^	0.2	0.001	0.017	0.001

WBC (×10^3^ *µ*L)	30	26.0	32.0	27.8	24.0	24.5	1.0	0.050	0.090	0.041
	60	9.7	9.5	9.2	8.8	9.3	0.7	0.101	0.115	0.211

HB (g dL^−1^)	30	5.9^b^	7.2^a^	7.2^a^	8.0^a^	7.4^a^	0.2	0.002	0.026	0.001
	60	6.4	6.3	5.4	7.0	6.7	0.3	0.554	0.426	0.731

Hct (%)	30	34.2^b^	41.5^a^	24.8^c^	24.2^c^	30.0^b^	1.4	0.001	0.067	0.003
	60	30.0^b^	29.8^b^	33.2^b^	24.4^c^	37.4^a^	1.0	0.001	0.040	0.002

MCH (pg cell^−1^)^1^	30	33.4	43.1	35.5	38.9	43.7	1.7	0.242	0.230	0.449
	60	23.8	25.5	22.3	28.7	27.4	1.2	0.481	0.629	0.089

MCV (fL)^2^	30	154.7^b^	246.1^a^	120.2^c^	116.5^c^	177.6^b^	10.3	0.001	0.152	0.365
	60	154.6^a^	116.8^ab^	136.4^ab^	100.6^b^	154.2^a^	4.3	0.001	0.545	0.001

MCHC (g dL^−1^)^3^	30	20.2^b^	17.8^b^	29.5^a^	33.4^a^	26.1^ab^	1.4	0.001	0.048	0.001
	60	20.1^ab^	21.2^ab^	17.4^b^	26.3^a^	18.9^ab^	2.9	0.015	0.941	0.222

^1^Mean cell volume (MCV) = Hct (%)/RBC (×10^6^ *µ*L) × 10. ^2^Mean cell hemoglobin (MCH) = Hb (g dL^−1^)/RBC (×10^6^ *µ*L) × 10. ^3^Mean cell hemoglobin concentration (MCHC) = Hb (g dL^−1^)/Hct (%). The values were expressed as mean ± pooled SE (*n* = 6). For each parameter, superscript letters denote significant (*P* < 0.05) differences between the values in each row. Abbreviations: RBC, red blood cell; WBC, white blood cell; HB, hemoglobin; Hct, hematocrit; MCH, mean cell hemoglobin; MCV, mean cell volume; MCHC, mean cell hemoglobin concentration.

**Table 8:** Influence of different levels of selenium nanoparticles dietary supplementation on serum biochemical parameters of Asian seabass (*Lates calcarifer*) (*n* = 6).

Parameters	Sampling time (day)	Control	SeNP 0.5	SeNP 1	SeNP 2	SeNP 4	Pooled SE	ANOVA	Linear	Quadratic
Glucose (mg dL^−1^)	30	24.9	26.3	27.0	25.9	24.6	0.5	0.511	0.423	0.304
	60	26.1	25.0	24.8	25.2	23.3	0.7	0.818	0.262	0.536

Calcium (mg dL^−1^)	30	31.3	30.7	32.1	30.3	31.2	0.6	0.905	0.907	0.966
	60	30.0	31.0	31.2	31.6	31.4	0.6	0.922	0.511	0.649

Cholesterol (mg dL^−1^)	30	103.1^a^	95.8^b^	91.8^b^	84.6^c^	84.9^c^	1.4	0.001	0.001	0.001
	60	102.4^a^	84.6^b^	80.0^b^	67.6^d^	75.2^c^	2.2	0.001	0.001	0.001

Triglyceride (mg dL^−1^)	30	125.9^a^	119.2^a^	104.9^b^	95.0^c^	94.2^c^	2.4	0.001	0.001	0.001
	60	127.0^a^	105.7^b^	88.6^c^	80.2^c^	69.9^d^	3.8	0.001	0.001	0.001

ALP (U L^−1^)	30	26.1^ab^	23.0^ab^	31.0^a^	32.6^a^	20.7^b^	1.3	0.004	0.290	0.005
	60	41.8^a^	21.2^b^	22.6^b^	20.3^b^	21.6^b^	5.5	0.001	0.020	0.001

AST (U L^−1^)	30	11.4^c^	27.8^a^	16.3^b^	13.6^c^	3.3^d^	1.5	0.001	0.001	0.001
	60	3.6^a^	2.6^b^	1.4^c^	1.5^c^	0.8^d^	0.2	0.001	0.001	0.001

ALT (U L^−1^)	30	2.0	1.3	1.3	2.0	1.3	0.1	0.131	0.368	0.643
	60	9.3^a^	3.2^b^	5.3^b^	3.8^b^	0.7^c^	0.5	0.001	0.001	0.001

LDH (U L^−1^)	30	293.7^a^	187.7^b^	184.6^b^	193.0^b^	111.0^c^	30.8	0.001	0.009	0.034
	60	172.1^a^	195.1^a^	173.2^a^	149.1^a^	58.8^b^	11.8	0.001	0.042	0.001

The values were expressed as mean ± pooled SE (*n* = 6). For each parameter, superscript letters denote significant (*P* < 0.05) differences between the values in each row. Abbreviations: ALP, alkaline phosphatase; AST, aspartate aminotransferase; ALT, alanine aminotransferase; LDH, lactate dehydrogenase.

## Data Availability

The datasets generated and/or analyzed during the current study are available from the corresponding author upon reasonable request.

## References

[1] Watanabe T., Kiron V., Satoh S. (1997). Trace minerals in fish nutrition. *Aquaculture*.

[2] Khademzade O., Kochanian P., Zakeri M., Alavi S. M. H., Mozanzadeh M. T. (2022). Oxidative stress-related semen quality and fertility in the male Arabian yellowfin sea bream (*Acanthopagrus arabicus*) fed a selenium nanoparticle-supplemented plant protein-rich diet. *Aquaculture Nutrition*.

[3] Izadpanah E., Saffari S., Keyvanshokooh S., Mozanzadeh M. T., Mousavi S. M., Pasha-Zanoosi H. (2022). Nano-selenium supplementation in plant protein-based diets changed thyroid hormones status and hepatic enzymes activity in *Acanthopagrus arabicus* female broodfish and their offspring. *Aquaculture Reports*.

[4] Saffari S., Keyvanshokooh S., Mozanzadeh M. T., Shahriari A. (2021). Effects of nano-selenium supplementation in plant protein-rich diet on reproductive performance and egg and larval quality of female Arabian yellowfin sea bream (*Acanthopagrus arabicus*). *Aquaculture Nutrition*.

[5] Papp L. V., Lu J., Holmgren A., Khanna K. K. (2007). From selenium to selenoproteins: synthesis, identity, and their role in human health. *Antioxidants & Redox Signaling*.

[6] Lin Y.-H., Shiau S.-Y. (2005). Dietary selenium requirements of juvenile grouper, *Epinephelus malabaricus*. *Aquaculture*.

[7] Abdolahpur Monikh F., Chupani L., Smerkova K. (2020). Engineered nanoselenium supplemented fish diet: toxicity comparison with ionic selenium and stability against particle dissolution, aggregation and release. *Environmental Science: Nano*.

[8] Dawood M. A. O., Basuini M. F. E., Yilmaz S. (2021). Selenium nanoparticles as a natural antioxidant and metabolic regulator in aquaculture: a review. *Antioxidants*.

[9] Khalil H. S., Maulu S., Verdegem M., Abdel-Tawwab M. (2023). Embracing nanotechnology for selenium application in aquafeeds. *Reviews in Aquaculture*.

[10] Sarkar B., Bhattacharjee S., Daware A., Tribedi P., Krishnani K. K., Minhas P. S. (2015). Selenium nanoparticles for stress-resilient fish and livestock. *Nanoscale Research Letters*.

[11] Li H., Zhang J., Wang T., Luo W., Zhou Q., Jiang G. (2008). Elemental selenium particles at nano-size (nano-Se) are more toxic to Medaka (*Oryzias latipes*) as a consequence of hyper-accumulation of selenium: a comparison with sodium selenite. *Aquatic Toxicology*.

[12] Saffari S., Keyvanshokooh S., Zakeri M., Johari S. A., Pasha-Zanoosi H., Mozanzadeh M. T. (2018). Effects of dietary organic, inorganic, and nanoparticulate selenium sources on growth, hemato-immunological, and serum biochemical parameters of common carp (*Cyprinus carpio*). *Fish Physiology and Biochemistry*.

[13] Neamat-Allah A. N. F., Mahmoud E. A., Abd El Hakim Y. (2019). Efficacy of dietary nano-selenium on growth, immune response, antioxidant, transcriptomic profile and resistance of Nile tilapia, *Oreochromis niloticus* against *Streptococcus iniae* infection. *Fish & Shellfish Immunology*.

[14] Naderi M., Keyvanshokooh S., Salati A. P., Ghaedi A. (2017). Effects of dietary vitamin E and selenium nanoparticles supplementation on acute stress responses in rainbow trout (*Oncorhynchus mykiss*) previously subjected to chronic stress. *Aquaculture*.

[15] Takahashi L. S., Biller-Takahashi J. D., Mansano C. F. M., Urbinati E. C., Gimbo R. Y., Saita M. V. (2017). Long-term organic selenium supplementation overcomes the trade-off between immune and antioxidant systems in pacu (*Piaractus mesopotamicus*). *Fish & Shellfish Immunology*.

[16] Mansour A. T.-E., Goda A. A., Omar E. A., Khalil H. S., Esteban M. (2017). Dietary supplementation of organic selenium improves growth, survival, antioxidant and immune status of meagre, *Argyrosomus regius*, juveniles. *Fish & Shellfish Immunology*.

[17] Dawood M. A. O., Koshio S., Zaineldin A. I. (2019). An evaluation of dietary selenium nanoparticles for red sea bream (*Pagrus major*) aquaculture: growth, tissue bioaccumulation, and antioxidative responses. *Environmental Science and Pollution Research*.

[18] Al-Deriny S. H., Dawood M. A. O., Elbialy Z. I., El-Tras W. F., Mohamed R. A. (2020). Selenium nanoparticles and spirulina alleviate growth performance, hemato-biochemical, immune-related genes, and heat shock protein in Nile Tilapia (*Oreochromis niloticus*). *Biological Trace Element Research*.

[19] Abd El-Kader M. F., El-Bab A. F. F., Shoukry M. (2020). Evaluating the possible feeding strategies of selenium nanoparticles on the growth rate and wellbeing of European seabass (*Dicentrarchus labrax*). *Aquaculture Reports*.

[20] Abd El-Kader M. F., El-Bab A. F. F., Abd-Elghany M. F., Abdel-Warith A.-W. A., Younis E. M., Dawood M. A. O. (2021). selenium nanoparticles act potentially on the growth performance, hemato-biochemical indices, antioxidative, and immune-related genes of European seabass (*Dicentrarchus labrax*). *Biological Trace Element Research*.

[21] Yan L., Johnson L. A. K. (2011). Selenium bioavailability from naturally produced high-selenium peas and oats in selenium-deficient rats. *Journal of Agricultural and Food Chemistry*.

[22] Wolffram S., Anliker E., Scharrer E. (1986). Uptake of selenate and selenite by isolated intestinal brush border membrane vesicles from pig, sheep, and rat. *Biological Trace Element Research*.

[23] Mozanzadeh M. T., Safari O., Oosooli R. (2021). The effect of salinity on growth performance, digestive and antioxidant enzymes, humoral immunity and stress indices in two euryhaline fish species: yellowfin seabream (*Acanthopagrus latus*) and Asian seabass (*Lates calcarifer*). *Aquaculture*.

[24] Tveteras R., Nystoyl R., Jory D. E. (2019). Global finfish production review and forecast. https://www.globalseafood.org/advocate/goal-2019-global-finfish-production-review-and-forecast.

[25] Ilham I., Siddik M. A. B., Fotedar R. (2016). Effects of organic selenium supplementation on growth, accumulation, haematology and histopathology of juvenile barramundi (*Lates calcarifer*) fed high soybean meal diets. *Biological Trace Element Research*.

[26] Longbaf Dezfouli M., Ghaedtaheri A., Keyvanshokooh S., Salati A. P., Mousavi S. M., Pasha-Zanoosi H. (2019). Combined or individual effects of dietary magnesium and selenium nanoparticles on growth performance, immunity, blood biochemistry and antioxidant status of Asian seabass (*Lates calcarifer*) reared in freshwater. *Aquaculture Nutrition*.

[27] Siddik M. A. B., Vatsos I. N., Rahman M. A., Pham H. D. (2022). Selenium-enriched spirulina (SeE-SP) enhance antioxidant response, immunity, and disease resistance in juvenile Asian seabass, *Lates calcarifer*. *Antioxidants*.

[28] Zhang J.-S., Gao X.-Y., Zhang L.-D., Bao Y.-P. (2001). Biological effects of a nano red elemental selenium. *BioFactors*.

[29] Fontagné-Dicharry S., Godin S., Liu H. (2015). Influence of the forms and levels of dietary selenium on antioxidant status and oxidative stress-related parameters in rainbow trout (*Oncorhynchus mykiss*) fry. *British Journal of Nutrition*.

[30] Kumar N., Krishnani K. K., Meena K. K., Gupta S. K., Singh N. P. (2017). Oxidative and cellular metabolic stress of *Oreochromis mossambicus* as biomarkers indicators of trace element contaminants. *Chemosphere*.

[31] Castro-Ruiz D., Mozanzadeh M. T., Fernández-Méndez C. (2019). Ontogeny of the digestive enzyme activity of the Amazonian pimelodid catfish *Pseudoplatystoma punctifer* (Castelnau, 1855). *Aquaculture*.

[32] Gisbert E., Nolasco H., Solovyev M. (2019). Towards the standardization of brush border purification and intestinal alkaline phosphatase quantification in fish with notes on other digestive enzymes. *Aquaculture*.

[33] Crane R. K., Boge G., Rigal A. (1979). Isolation of brush border membranes in vesicular form from the intestinal spiral valve of the small dogfish (*Scyliorhinus canicula*). *Biochimica et Biophysica Acta (BBA)-Biomembranes*.

[34] Bradford M. M. (1976). A rapid and sensitive method for the quantitation of microgram quantities of protein utilizing the principle of protein-dye binding. *Analytical Biochemistry*.

[35] Bergmeyer H. U. (1974). *Methods of Enzymatic Analysis*.

[36] Hummel B. C. (1959). A modified spectrophotometric determination of chymotrypsin, trypsin, and thrombin. *Canadian Journal of Biochemistry and Physiology*.

[37] Folin O., Ciocalteu V. (1929). Enzymatic assay of protease using casein as a substrate. *Journal of Biological Chemistry*.

[38] Bessey O. A., Lowry O. H., Brock M. J. (1946). A method for the rapid determination of alkaline phosphatase with five cubic millimeters of serum. *Journal of Biological Chemistry*.

[39] Bernfeld P. (1955). Amylases, *α* and *β*. *Methods in Enzymology*.

[40] Tietz N. W., Fiereck E. A. (1966). A specific method for serum lipase determination. *Clinica Chimica Acta*.

[41] Aebi H., Bergmeyer H. V. (1974). Catalase. *Methods in Enzymatic Analysis*.

[42] McCord J. M., Fridovich I. (1969). Superoxide dismutase. *Journal of Biological Chemistry*.

[43] Beutler E., Duron O., Kelly B. M. (1963). Improved method for the determination of blood glutathione. *Journal of Laboratory and Clinical Medicine*.

[44] Blaxhall P. C., Daisley K. W. (1973). Routine haematological methods for use with fish blood. *Journal of Fish Biology*.

[45] Dacie J. V., Lewis S. M. (2001). *Practical Hematology*.

[46] Siwicki A. K., Anderson D. P., Rumsey G. L. (1994). Dietary intake of immunostimulants by rainbow trout affects non-specific immunity and protection against furunculosis. *Veterinary Immunology and Immunopathology*.

[47] Ellis A. E. (1990). Serum antiproteases in fish and lysozyme assays. *Techniques in Fish Immunology*.

[48] Brata O. (1993). *Veterinary Clinical Immunology Laboratory 2*.

[49] Ghanei-Motlagh R., Mohammadian T., Gharibi D. (2021). Quorum quenching probiotics modulated digestive enzymes activity, growth performance, gut microflora, haemato-biochemical parameters and resistance against *Vibrio harveyi* in Asian seabass (*Lates calcarifer*). *Aquaculture*.

[50] Abbaszadeh A., Yavari V., Hoseini S. J., Nafisi M., Torfi Mozanzadeh M. (2019). Effects of different carbon sources and dietary protein levels in a biofloc system on growth performance, immune response against white spot syndrome virus infection and cathepsin L gene expression of *Litopenaeus vannamei*. *Aquaculture Research*.

[51] Moges F. D., Patel P., Parashar S. K. S., Das B. (2020). Mechanistic insights into diverse nano-based strategies for aquaculture enhancement: a holistic review. *Aquaculture*.

[52] Wang L., Zhang X., Wu L., Liu Q., Zhang D., Yin J. (2018). Expression of selenoprotein genes in muscle is crucial for the growth of rainbow trout (*Oncorhynchus mykiss*) fed diets supplemented with selenium yeast. *Aquaculture*.

[53] Han D., Xie S., Liu M. (2011). The effects of dietary selenium on growth performances, oxidative stress and tissue selenium concentration of gibel carp (*Carassius auratus gibelio*). *Aquaculture Nutrition*.

[54] Wang, Yan X., Fu L. (2013). Effect of selenium nanoparticles with different sizes in primary cultured intestinal epithelial cells of crucian carp, *Carassius auratus gibelio*. *International Journal of Nanomedicine*.

[55] Izquierdo M. S., Ghrab W., Roo J. (2017). Organic, inorganic and nanoparticles of Se, Zn and Mn in early weaning diets for gilthead seabream (*Sparus aurata;* Linnaeus, 1758). *Aquaculture Research*.

[56] Kianersi F., Safahieh A. R., Salamat N., Salati A. P., Houshand H. (2021). Effect of sodium selenite and selenium nanoparticles on biochemical parameters of muscle, serum, antioxidant defense and exposure to mercury chloride in *Acanthopagrus latus*. *Iranian Scientific Fisheries Journal*.

[57] Ashouri S., Keyvanshokooh S., Salati A. P., Johari S. A., Pasha-Zanoosi H. (2015). Effects of different levels of dietary selenium nanoparticles on growth performance, muscle composition, blood biochemical profiles and antioxidant status of common carp (*Cyprinus carpio*). *Aquaculture*.

[58] El-Sharawy M. E., Hamouda M., Soliman A. A. (2021). Selenium nanoparticles are required for the optimum growth behavior, antioxidative capacity, and liver wellbeing of striped catfish (*Pangasianodon hypophthalmus*). *Saudi Journal of Biological Sciences*.

[59] Shenkin A. (2006). Micronutrients in health and disease. *Postgraduate Medical Journal*.

[60] Iqbal S., Atique U., Mughal M. S. (2017). Effect of selenium incorporated in feed on the hematological profile of Tilapia (*Oreochromis niloticus*). *Journal of Aquaculture Research & Development*.

[61] Said R. M., Nassar S. E., Alaidaroos B. A. (2023). Impacts of dietary selenium nanoparticles from *Spirulina platensis* on growth performance, physio-biochemical components and alleviating effect against cadmium toxicity in pacific white shrimp *Litopenaeus vannamei*. *Catalysts*.

[62] Eissa E.-S. H., Bazina W. K., Abd El-Aziz Y. M. (2023). Nano-selenium impacts on growth performance, digestive enzymes, antioxidant, immune resistance and histopathological scores of Nile tilapia, *Oreochromis niloticus* against *Aspergillus flavus* infection. *Aquaculture International*.

[63] Nugroho R. A., Fotedar R. (2015). Effects of dietary organic selenium on immune responses, total selenium accumulation and digestive system health of marron, *Cherax cainii* (Austin, 2002). *Aquaculture Research*.

[64] Pour H. D., Mousavi S. M., Zakeri M., Keyvanshokooh S. (2021). Synergistic effects of selenium and magnesium nanoparticles on growth, digestive enzymes, some serum biochemical parameters and immunity of Asian sea bass (*Lates calcarifer*). *Biological Trace Element Research*.

[65] Rotruck J. T., Pope A. L., Ganther H. E., Swanson A. B., Hafeman D. G., Hoekstra W. G. (1973). Selenium: biochemical role as a component of glutathione peroxidase. *Science*.

[66] Mehdi Y., Hornick J.-L., Istasse L., Dufrasne I. (2013). Selenium in the environment, metabolism and involvement in body functions. *Molecules*.

[67] Aksnes A., Njaa L. R. (1981). Catalase, glutathione peroxidase and superoxide dismutase in different fish species. *Comparative Biochemistry and Physiology Part B: Comparative Biochemistry*.

[68] Dawood M. A. O., Zommara M., Eweedah N. M., Helal A. I. (2020). The evaluation of growth performance, blood health, oxidative status and immune-related gene expression in Nile tilapia (*Oreochromis niloticus*) fed dietary nanoselenium spheres produced by lactic acid bacteria. *Aquaculture*.

[69] Bai Z., Ren T., Han Y., Hu Y., Schohel M. R., Jiang Z. (2019). Effect of dietary bio-fermented selenium on growth performance, nonspecific immune enzyme, proximate composition and bioaccumulation of zebrafish (*Danio rerio*). *Aquaculture Reports*.

[70] Le K. T., Fotedar R., Partridge G. (2014). Selenium and vitamin E interaction in the nutrition of yellow kingfish (*Seriola lalandi*): physiological and immune responses. *Aquaculture Nutrition*.

[71] Naderi M., Keyvanshokooh S., Ghaedi A., Salati A. P. (2017). Combined or individual effects of dietary vitamin E and selenium nanoparticles on humoral immune status and serum parameters of rainbow trout (*Oncorhynchus mykiss*) under high stocking density. *Aquaculture*.

[72] Zhou X., Wang Y., Gu Q., Li W. (2009). Effects of different dietary selenium sources (selenium nanoparticle and selenomethionine) on growth performance, muscle composition and glutathione peroxidase enzyme activity of crucian carp (*Carassius auratus gibelio*). *Aquaculture*.

[73] Molinero P., Osuna C., Guerrero J. M. (1995). Type II thyroxine 5′-deiodinase in the rat thymus. *Journal of Endocrinology*.

[74] Le K. T., Fotedar R. (2014). Bioavailability of selenium from different dietary sources in yellowtail kingfish (*Seriola lalandi*). *Aquaculture*.

[75] Magnadottir B. (2006). Innate immunity of fish (overview). *Fish & Shellfish Immunology*.

[76] Khan K. U., Zuberi A., Nazir S., Fernandes J. B. K., Jamil Z., Sarwar H. (2016). Effects of dietary selenium nanoparticles on physiological and biochemical aspects of juvenile Tor putitora. *Turkish Journal of Zoology*.

[77] Uribe C., Folch H., Enriquez R., Moran G. (2011). Innate and adaptive immunity in teleost fish: a review. *Veterinární medicína*.

[78] Liu S., Yu H., Li P. (2022). Dietary nano-selenium alleviated intestinal damage of juvenile grass carp (*Ctenopharyngodon idella*) induced by high-fat diet: insight from intestinal morphology, tight junction, inflammation, anti-oxidization and intestinal microbiota. *Animal Nutrition*.

[79] Witeska M., Kondera E., Ługowska K., Bojarski B. (2022). Hematological methods in fish—not only for beginners. *Aquaculture*.

[80] Pighetti G. M., Eskew M. L., Reddy C. C., Sordillo L. M. (1998). Selenium and vitamin E deficiency impair transferrin receptor internalization but not IL-2, IL-2 receptor, or transferrin receptor expression. *Journal of Leukocyte Biology*.

[81] Biller-Takahashi J. D., Takahashi L. S., Mingatto F. E., Urbinati E. C. (2015). The immune system is limited by oxidative stress: dietary selenium promotes optimal antioxidative status and greatest immune defense in pacu *Piaractus mesopotamicus*. *Fish & Shellfish Immunology*.

[82] Ahmad N., Hussain S. M., Azam S. M. (2024). Effects of Se nanoparticles supplementation on growth performance, hematological parameters and nutrient digestibility of *Labeo rohita* fingerling fed sunflower meal based diet. *Brazilian Journal of Biology*.

[83] Al-Dwairi A., Brown A. R., Pabona J. M. P. (2014). Enhanced gastrointestinal expression of cytosolic malic enzyme (ME1) induces intestinal and liver lipogenic gene expression and intestinal cell proliferation in mice. *PLOS ONE*.

[84] Dhingra S., Bansal M. P. (2006). Attenuation of LDL receptor gene expression by selenium deficiency during hypercholesterolemia. *Molecular and Cellular Biochemistry*.

[85] Dhingra S., Bansal M. P. (2006). Modulation of hypercholesterolemia-induced alterations in apolipoprotein B and HMG-CoA reductase expression by selenium supplementation. *Chemico-Biological Interactions*.

[86] Yang K.-C., Lee L.-T., Lee Y.-S., Huang H.-Y., Chen C.-Y., Huang K.-C. (2010). Serum selenium concentration is associated with metabolic factors in the elderly: a cross-sectional study. *Nutrition & Metabolism*.

[87] Çiçek S., Ozogul F. (2021). Effects of selenium nanoparticles on growth performance, hematological, serum biochemical parameters, and antioxidant status in fish. *Animal Feed Science and Technology*.

[88] Hao X., Ling Q., Hong F. (2014). Effects of dietary selenium on the pathological changes and oxidative stress in loach (*Paramisgurnus dabryanus*). *Fish Physiology and Biochemistry*.

[89] Ogunfowora L. A., Iwuozor K. O., Ighalo J. O., Igwegbe C. A. (2021). Trends in the treatment of aquaculture effluents using nanotechnology. *Cleaner Materials*.

[90] Fajardo C., Martinez-Rodriguez G., Blasco J., Mancera J. M., Thomas B., De Donato M. (2022). Nanotechnology in aquaculture: applications, perspectives and regulatory challenges. *Aquaculture and Fisheries*.

[91] Ahmadi-Noorbakhsh S., Mirabzadeh Ardakani E., Sadighi J. (2021). Guideline for the care and use of laboratory animals in Iran. *Lab Animal*.

